# Hundred Fifty Years of Herbarium Collections Provide a Reliable Resource of Volatile Terpenoid Profiles Showing Strong Species Effect in Four Medicinal Species of *Salvia* Across the Mediterranean

**DOI:** 10.3389/fpls.2018.01877

**Published:** 2018-12-18

**Authors:** Isa Jafari Foutami, Trine Mariager, Riikka Rinnan, Christopher James Barnes, Nina Rønsted

**Affiliations:** ^1^Gorgan University of Agricultural Sciences and Natural Resources, Gorgan, Iran; ^2^Terrestrial Ecology Section, Department of Biology, University of Copenhagen, Copenhagen, Denmark; ^3^Natural History Museum of Denmark, University of Copenhagen, Copenhagen, Denmark

**Keywords:** age, altitude, collections, GC-MS, geography, herbarium, *Salvia*, terpene

## Abstract

Herbarium samples are increasingly being recognized for their potential in answering a wide range of research questions. However, the suitability of herbarium samples for chemical analysis is largely unexplored as they are thought to be too degraded. The aim of this study was to explore terpenoid profiles across time and geographic space for four medicinal species of *Salvia* across the Mediterranean to assess the suitability of using herbarium specimens in chemical analyses. Herbarium samples of *Salvia aethiopis, S. multicaulis, S. officinalis*, and *S. sclarea* collected over 150 years across the Mediterranean were compared to modern samples using both targeted and untargeted gas chromatography-mass spectrometry analysis of terpene profiles. There was no effect of collection year on chemical composition, although the total concentration of the 20 assessed standards and two individual standards significantly decreased over time. Instead, chemical profiles were defined by species, with strong species effects identified on both the targeted and untargeted chemical composition. Geographic variation was a factor in regulating the untargeted chemical compositions, suggesting some underlying environmental effects. However, there was no effect of sample altitude on either the targeted or untargeted chemical compositions. Chemical composition of four *Salvia* species are predominantly defined by species, and there was a substantially smaller effect of year of sampling. Given these results herbarium collections may well represent a considerably underused resource for chemical analyses that can benefit biodiversity and other studies.

## Introduction

Herbarium collections are increasingly being recognized as a unique, verifiable, and underused resource of big data across time and space for a variety of research questions (Lavoie, 2012; Funk, 2014; Rønsted et al., 2017; Cardoso et al., 2018; James et al., 2018). Herbarium collections offer an easily accessible source of specimens for a plethora of research questions compared to field expeditions (Bebber et al., 2010; Hardion et al., 2014; Xu et al., 2015). Additionally, herbarium specimens are records in time and space and represent several 100 years of collection history across the globe, including species rare and extinct or from now gone habitats (Silva et al., 2017) that provide possibilities simply not available using only modern samples.

Collections provide a time window into the past allowing exploration of changes in composition of floras (Calinger, 2015), distribution of invasive weeds or threatened species (Rivers et al., 2011; Martin et al., 2016), changes in flowering times (Davis et al., 2015) or leaf-out times (Everill et al., 2014) in response to climate change as well as to model predictions of future trends (James et al., 2018).

As the technical difficulties of extracting high quality DNA from historical materials are being overcome (Bieker and Martin, 2018), herbarium materials are also increasingly being used in genomic studies at all scales, from populations to phylogenies (Kuzmina et al., 2017; Bieker and Martin, 2018) as well as to study domestication history (da Fonseca et al., 2015) or plant pathogens (Yoshida et al., 2014).

Likewise, herbarium materials could be a resource of chemical data potentially relevant for chemotaxonomy (Cook et al., 2009; Yilmaz et al., 2012), chemical ecology (Zangerl and Berenbaum, 2005), environmental bioindicators (Foan et al., 2010; Monforte et al., 2015) as well as drug discovery and authentication (Saslis-Lagoudakis et al., 2015; Rønsted et al., 2017).

However, the exploration of herbarium material for plant metabolite data is comparably understudied and the reliability of chemical data from herbarium samples is uncertain due to the expectation that plant specialized metabolites may not be well-preserved over longer time scales. Whereas alkaloids for example are generally considered highly stable (Cook et al., 2009; Dewick, 2009; Yilmaz et al., 2012), low molecular weight terpenoids tend to be both volatile and thermolabile and may be easily oxidized or hydrolyzed altering the chemical composition of the plant material dependent on the conditions during processing and storage of the plant material (Turek and Stinzing, 2013; Tasca et al., 2018).

It is therefore essential for any study using chemical data extracted from herbarium materials to test for potential age effects on results in order to verify interpretations (Jafari et al., 2018). Only few studies to date have systematically tested the stability of different compound classes in historical herbarium samples. An early study by Eloff (1999) showed no sample age effect on antibacterial activity of herbarium samples of *Combretum erythophyllum* (Burch.) Sond. and *Helichrysum pedunculatum* Hilliard & B.L. Burtt over almost 100 years and only minor differences were observed in chemical composition of flavonoids and terpenoids using thin layer chromatography (TLC). Using proton nuclear magnetic resonance (^1H^NMR) spectroscopic metabolite screening, Jafari et al. (2018) found no significant difference in metabolite profiles of recently dried and up to 25 year old fungi specimens. Cook et al. (2009) found no age effect on toxic diterpenoid alkaloid composition of *Delphinium occidentale* S. Watson from up to 100 year old herbarium specimens and Yilmaz et al. (2012) found no significant degradation of quinine alkaloids in 50–150 years old historical Cinchonae bark samples. Likewise, a study by Zangerl and Berenbaum (2005) found no sampling age effect on furanocoumarins in the phototoxic invasive *Pastinaca sativa* L. over 150 years. Consequently, these previous studies support the idea that herbarium specimens provide a significant untapped resource of chemical data in addition to data on distribution and morphological traits over time and space.

Plant specialized metabolites are selected through evolution for their biological activities and express some degree of phylogenetic clustering of different compound classes (e.g., Hegnauer, 1962–1973; Ehrlich and Raven, 1964; Rønsted et al., 2012). However, natural variation in plant chemical composition is common and attributed to both biotic (e.g., herbivory and microorganisms) and abiotic environmental conditions as well as potentially differential local chemotypes (Wink, 2003; Moore et al., 2014). The relative importance of different drivers of chemical diversity is an outstanding puzzle, but recent attention has focused on altitude as an explanatory parameter (Russo et al., 2013; Mahzooni-Kachapi et al., 2014; Senica et al., 2017; Pandey et al., 2018). In addition to abiotic components, correlation of chemical variation with altitude is hypothesized to be related to variable biotic pressure from herbivory and microbial communities (Abdala-Roberts et al., 2016; Maldonado et al., 2017; Pandey et al., 2018). Herbarium collections along with their associated data can provide specimens of verified geographical and altitudinal origin for further studies.

In this work, we tested the suitability of using herbarium specimens in plant chemical studies by assessing targeted and untargeted chemical variation of four *Salvia* species. Samples ranged from modern collections to 150 year old herbarium collections. We had three main objectives. (1) Our first objective was to investigate whether we could observe significant chemical variation between *Salvia* species using herbarium specimens, and compare the results to other studies that have used modern material. (2) Our second objective was to investigate age effects on *Salvia* chemical variation, testing for changes in chemical composition and diversity associated with increasing sample age. (3) Our final objective was to assess whether herbarium specimens could be used in answering ecological questions on *Salvia* chemical composition variation and diversity across altitudes and geographical regions in the four *Salvia* species.

## Materials and Methods

### Plant Material Sampling Strategy and Study Design

**Figure 1:**
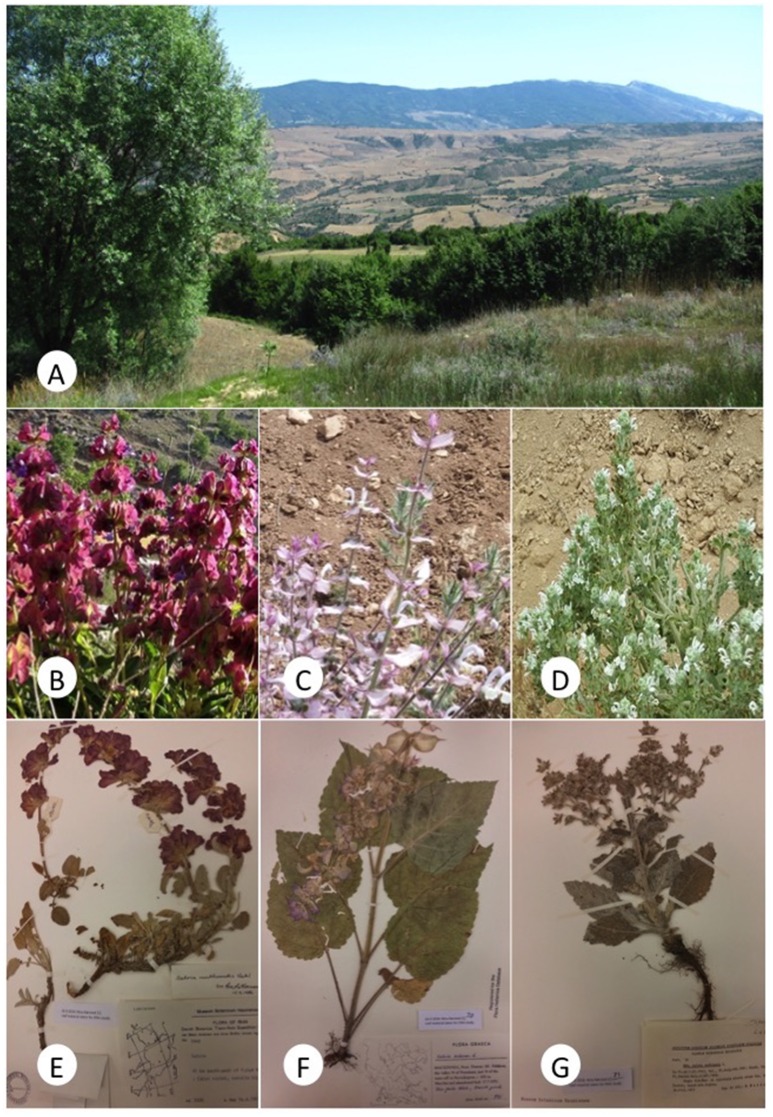
**(A)** Habitat of *Salvia*, Mazandaran province, Iran. **(B)** *S. multicaulis*. **(C)** *S. sclarea*. **(D)** *S. aethiopis*. **(E)** *S. multicaulis* specimen no. 13. **(F)** *S. sclarea* specimen no. 30. **(G)** *S. aethiopis* specimen no. 31.

As a case study, we selected three Iranian medicinal *Salvia* species, which are all locally rare, and exhibiting a diversity of distributional and altitudinal ranges (Jafari Foutami and Akbarlou, 2017; Figure 1). For each of the four species we attempted to sample across the geographical distribution of the species. *Salvia sclarea* L. (clary sage; *N* = 10) is native to the Northern Mediterranean but has also become a weed outside its native range including North America, where it is called European sage (Ghani et al., 2010; Dickinson and Royer, 2014). *Salvia multicaulis* Vahl (syn. *S. acetabulosa* L.; *N* = 8) is native to Turkey and bordering countries (Tepe et al., 2004). *Salvia aethiopis* L. (*N* = 4) is naturally occurring in the Northern Mediterranean but has become a noxious weed in North America, where it is referred to as Mediterranean sage (Chalchat et al., 2001; Dickinson and Royer, 2014). Additionally, we included *S. officinalis* (*N* = 12) allowing for comparison with extensive literature and verified reference material adhering to the European Pharmacopeia standards (Council of Europe, 2014).

**Figure 2:**
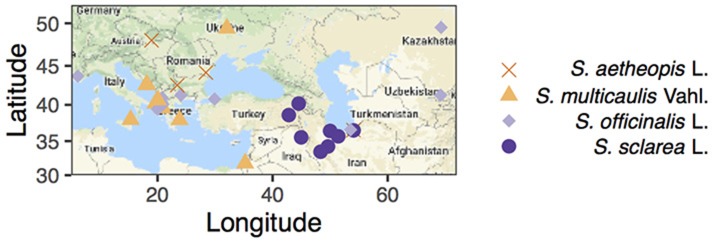
Map of collections with shape and color representing species (note four samples for which we had no geographic information are excluded from the map). Maps were constructed using ggmap (Kahle and Wickham, 2013).

*Salvia officinalis* L. Ph.Eur 8.3. quality leaf reference material was obtained from Alfred Galke GmbH, Germany. Fresh samples of *S. aethiopis, S. multicaulis* and *S. sclarea* were collected in Iran in 2017 and air-dried at room temperature. Herbarium material of *S. aethiopis, S. multicaulis, S. officinalis*, and *S. sclarea* was obtained from Herbarium C of the Natural History Museum of Denmark, University of Copenhagen spanning 150 years of collecting across the Mediterranean (Figure 2).

Taxonomic identity of specimen was confirmed to species morphologically by Isa Jafari Foutami. Collection year was recorded from labels and are listed in Table 1 together with altitude and GPS coordinates. However, in most cases, GPS coordinates and in a few cases altitude had to be inferred from the locality description on the labels. Details of all plant materials are listed in Table 1.

**Table 1:** Details of *Salvia* materials included in this study.

**Code**	**Taxon**	**Collection year**	**Collector and voucher details**	**Altitude**	**Country**	**Locality**	**Latitude**	**Longitude**
1	*S. officinalis* L.	1993	A. Strid et al. 35900 (C)	600 m	Greece	Ioannina, Dodonis, Aristiti	39.95	20.65
2	*S. officinalis* L.	1980	P. Hartvig & R. Franzén 9075 (C)	500–650 m	Greece	Ioannina, Konitsis, Mt. Timfi, Aoös Gorge	39.58^a^	20.80^a^
3	*S. officinalis* L.	1966	Bregnhøj Larsen (C)	Unknown	Greece	Hymettus, Athen	37.97^a^	23.82^a^
4	*S. officinalis* L.	1906	V. Tyranovetz 4843 (C)	Unknown	Ukraine	Cherkasy	49.43^a^	32.05^a^
5	*S. officinalis* L.	1980	F. Krendl (C)	1300 m	Albania	Vlora, Mali, Mt Çika	40.20^a^	19.63^a^
6	*S. officinalis* L.	1973	N. Kaae (C)	Near sea level	Croatia	Dubrovnik, Lapad	42.63^a^	18.10^a^
7	*S. officinalis* L.	1980	Endlicher 3597–15 (C)		Cultivated	Botanical garden C (cultivated)		
8	*S. officinalis* L.	1975	O.B. Lyshede (C)	750 m^a^	Israel	Jerusalem	31.78^a^	35.22^a^
9	*S. officinalis* L.	1973	N. Kaae (C)	400 m	Croatia	Dubrovnik, Mount Srd	42.65^a^	18.12^a^
10	*S. officinalis* L.	1901	A. Toepffer (C)		Unknown	Flora megapolitana. Possibly cultivated.		
11	*S. officinalis* L.	1972	N. Kaae (C)	400 m^a^	Yugoslavia	Dubrovnik, Mount Srd	42.65^a^	18.12^a^
12	*S. officinalis* L.	1982	P. Ølgaard (C)	417 m^a^	Italy	Sicily, Mandanici	38.00^a^	15.32^a^
13	*S. multicaulis* Vahl	1973	J.S. Andersen and A.G. Jensen 7040 (C)	2,400 m	Iran	Fulad Makkaleh	35.68^a^	51.42^a^
14	*S. multicaulis* Vahl	1971	K. H. Rechinger 39461 (C)	1,100–1,300 m	Iran	Qazvin	36.45^a^	50.00^a^
15	*S. multicaulis* Vahl	1862	E. Bourgeau (C)		Armenia	Gumusch-Khani	40.17^a^	44.52^a^
16	*S. multicaulis* Vahl	1955	H. Helbeak 320 (C)	800 m^a^	Iraq	Jarmo	35.53^a^	44.95^a^
17	*S. multicaulis* Vahl	1937	M. Køie (C)	2,200 m	Iran	Bordsch, Lorestan/ Makazi Province	34.23^a^	49.63^a^
18	*S. multicaulis* Vahl	1937	M. Køie 750 (C)	2,100 m^a^	Iran	Khorramabad	33.48^a^	48.35^a^
19	*S. multicaulis* Vahl	1870	E. Cosson (C)	1,730 m^a^	Turkey	Yede Kilissa, Van	38.63^a^	42.82^a^
20	*S. multicaulis* Vahl	1955	Helbeak 865 (C)	800 m^a^	Iraq	Jarmo	35.53^a^	44.95^a^
21	*S. sclarea* L.	1963	V. Goloskokov (C)	450 m^a^	Kazakhstan	Alatau transiliensis, Kzyl-Saj	49.53^a^	69.28^a^
22	*S. sclarea* L.	1986	T.S. Ellsa, D. Murray & L. Newcombe 9756 (C)	1,700–2,000 m	Uzbekistan	Tashkent, Oblast, Karzhantau Ridge	41.27^a^	69.22^a^
23	*S. sclarea* L.	1962	David & Coode 36237 (C)	50 m	Turkey	Izmit-Gebse	40.77^a^	29.92^a^
24	*S. sclarea* L.	1976	W. Greuter 13847 (C)	1,000 m	Macedonia	Trpejca	40.95^a^	20.78^a^
25	*S. sclarea* L.	1979	P. Frost-Olsen 2064 (C)	477 m^a^	Serbia	Srbija, Kosmet, Orahovac	40.38^a^	20.65^a^
26	*S. sclarea* L.	1973	B. de Retz 67584 (C)	500 m	France	Var, Baudinard	43.72^a^	6.13^a^
27	*S. sclarea* L.	1976	P. Hartvig, C. Baden et al. (C)	920 m	Greece	Ioannina, Konitsa, Mt. Trapezitsa	40.05^a^	20.75^a^
28	*S. sclarea* L.	1979	A. Hansen 581(C)	34 m^a^	Greece	Corfu, Agios Nikolaos	38.43^a^	20.00^a^
29	*S. sclarea* L.	1975	P. Hartvig & O. Seberg 4860 (C)	1,275–1,325 m	Greece	Ioannina, Mt. Smolikas, Samarina-Armata Road	40.08^a^	20.92^a^
30	*S. sclarea* L.	1970	A. Strid 931 (C)	620 m	Greece	Drama, Mt. Falakro, Prosotsani	41.28^a^	24.08^a^
31	*S. aethiopis* L.	1973	M. Markova (C)	700 m	Bulgaria	Vitosha, Pancharevo, Urban Sophia	42.57^a^	23.43^a^
32	*S. aethiopis* L.	1963	A. Hansen (C)	23 m^a^	Romania	Murfatlar, Constanta	44.17^a^	28.40^a^
33	*S. aethiopis* L.	1958	M. Deyl, J. Nitka, B. Vytous (C)	150 m	Slovakia	Sahy	48.05^a^	18.95^a^
34	*S. aethiopis* L.	1971		Sea level	Alps	Cultivated in C		
35	*S. aethiopis* L.	2017	I. Jafari Foutami 3512, S521 (IMPH)	1,956 m	Iran	Hezar jerib, Mazandaran province	36.55	54.05
36	*S. multicaulis* Vahl	2017	I. Jafari Foutami 3621, S512 (IMPH)	2,174 m	Iran	Hezar jerib, Mazandaran province	36.52	54.08
37	*S. sclarea* L.	2017	I. Jafari Foutami 2150, S530 (IMPH)	1,232 m	Iran	Hezar jerib, Mazandaran province	36.60	53.55
38	*S. officinalis* L.	2018	Alfred Galke GmbH, Germany, Ph.Eur 8.3 reference material	Unknown	Albania			

a *Altitude and/or GPS data estimated from locality description on labels*.

### Terpenoid Extraction and Analysis

Twenty-Five Milligram leaf material was ground using a mortar and pestle under liquid N2 and transferred to a vial (Supelco, Pennsylvania, USA). Seven Hundred Microliter analytical grade hexane (Sigma-Aldrich, Denmark) was added, the sample was vortexed for 10 s and then incubated on a shaker at 37°C for 2 h. Samples were then vortexed again for 10 s and left for 24 h to allow tissue to settle. Approximately 200 μL of the hexane extract was transferred into a weighed glass GC vial with a limited volume insert and kept at −20°C until GC-MS analysis. The remainder of the sample was left uncapped at 65°C until the solvent was evaporated. Vials were reweighed to obtain the dry mass of leaf material. Samples were analyzed in triplicate.

GC–MS analyses were carried out using an Agilent 6890N Gas Chromatograph equipped with a split/splitless injector (200°C), a HP-5MS capillary column (30 m × 0.25 mm; film thickness 0.25 μm), and coupled with an Agilent 5975 MS Detector (MSD), operating in the electron impact (EI) mode at 70 eV. The carrier gas was helium (1.0 mL/min), and the oven temperature was programmed to increase from 60°C to 280°C at a rate of 3°C/min. The injected volume was 2 μL.

Chromatograms were analyzed both as untargeted data using tentative identifications based on the mass spectra in the NIST 8.0 library (National Institute of Standards and Technology, Gaithersburg, MD, USA) and as targeted data using 20 standard compounds.

Commercially available pure chemical certified reference material standards of common *Salvia* constituents (Russo et al., 2013; Hatipoglu et al., 2016; Craft et al., 2017) were obtained from Sigma-Aldrich, Germany (Table 2). Purity of the commercial standards was not investigated experimentally and the approach does therefore not guarantee no degradation of the standards could have happened. However, for the targeted approach, all samples were analyzed using the same standards.

**Table 2:** Average percentage (standard deviation) of standard compounds in targeted dataset (monoterpene hydrocarbons, oxygenated monoterpenes, and sesquiterpenes) compared with Hatipoglu et al. (2016) and Raal et al. (2007).

**Compound number and type**		**Retention time (minutes)**	***Salvia aethiopis***		***Salvia multicaulis***		***Salvia officinalis***		***Salvia sclarea***	
			**This study**	**Hatipoglu**	**This study**	**Hatipoglu**	**This study**	**Raal**	**This study**	**Hatipoglu**
1	α-Pinene	8.32	0.42 (0.93)	0.25	**12.69 (22.40)**	3.07	0.74 (0.43)	**≤6.4%**	0.02 (0.06)	0.44
2	Camphene	8.61	0.04 (0.08)	0	**4.70 (3.63)**	1.33	**2.24 (1.95)**	**≤7.1%**	0.02 (0.05)	0.95
3	β-Pinene	9.27	0.25 (0.11)	0	0.06 (0.07)	**13.49**	0.04 (0.04)	≤ 4.9%	0.15 (0.08)	0.29
4	Myrcene	9.65	0.44 (0.20)	1.84	0.16 (0.25)	0.11	0.06 (0.06)	≤ 4.2%	0.38 (0.14)	0
5	α-Phellandrene	9.94	0.09 (0.09)	0	0.03 (0.02)	0	0.02 (0.02)	≤ 0.1%	0.04 (0.03)	0
6	3-Carene	10.09	0.47 (0.23)	–	0.05 (0.04)	–	0.06 (0.07)	–	0.39 (0.15)	–
7	Limonene	10.50	0	0	0.05 (0.04)	0.37	0.04 (0.04)	0.0%	0	0
9	α-Oximene	10.97	0.21 (0.11)	–	0.02 (0.02)	–	0.03 (0.03)	–	0.17 (0.07)	–
10	⋎-Terpinene	11.22	0.22 (0.11)	–	0.03 (0.02)	–	0.03 (0.02)	≤ 0.7%	0.17 (0.05)	–
11	Terpinolene	11.90	**1.18 (0.49)**	0	0.84 (0.63)	0.47	**4.33 (3.81)**	≤ 0.5%	0.71 (0.29)	0.44
14	*p*-Cymene	16.17	1.02 (0.73)	0	**15.74 (10.29)**	0.25	**15.83 (17.79)**	≤ 1.0%	1.23 (1.04)	5.90
Total monoterpene hydrocarbons			4.35 (1.38)	2.09%	**34.38 (25.64)**	19.18	**23.42 (18.36)**	–	3.27 (1.36)	11.25
8	Eucalyptol	10.56	0	–	0.54 (0.49)	–	0.49 (0.39)	0.0	0	–
12	Linalool	12.15	0.24 (0.13)	0.55	0.03 (0.02)	0	0.04 (0.02)	–	0.21 (0.11)	0.29
13	Borneol	13.44	**17.07 (2.25)**	0.23	**44.91 (23.85)**	**6.07**	**49.82 (24.59)**	**≤11.8**	**11.69 (2.31)**	2.43
15	Bornyl acetate	16.22	0.24 (0.12)	0.18	0.53 (0.45)	0	0.09 (0.06)	**≤7.8**	0.20 (0.08)	2.21
20	1R-(+)-Camphor	24.37	**71.65 (10.81)**	0.23	**19.37 (17.30)**	**13.42**	**25.83 (24.66)**	**≤29.8**	**83.47 (2.89)**	0.34
Total oxygenated monoterpenes			**89.20 (11.82)**	12.49	**65.39 (25.69)**	39.41	**76.28 (18.40)**	–	**95.57 (1.71)**	13.76
16	β-Caryophyllene	19,24	**3.86 (7.85)**	1.26	0.04 (0.02)	2.87	0.05 (0.05)	**≤7.5**	0.32 (0.20)	2.58
17	Aromandendrene	19.65	0.36 (0.16)	0.63	0.07 (0.04)	0.14	0.12 (0.13)	–	0.30 (0.11)	0.29
18	α-Humulene (α -caryophyllene)	19.99	**1.77 (3.52)**	0.68	0.07 (0.14)	2.54	0.08 (0.11)	**≤8.5**	0.14 (0.05)	1.47
19	Nerolidol	22.31	0.46 (0.16)	0.18	0.05 (0.04)	0.16	0.05 (0.04)	–	0.40 (0.15)	0
Total sesquiterpenes			6.45 (11.14)	85.42	0.23 (0.15)	41.41	0.30 (0.24)	–	1.16 (0.38)	**74.99**
Total percentage of terpenoids			100%	100%	100%	100%	100%	–	100%	100%

*Major compounds in bold*.

A dilution series of the standard compounds in hexane was made and analyzed as samples: linear regression was used to calculate the concentrations of samples (*R*^2^ ≥ 0.886 for individual compounds). The set of standards included monoterpene hydrocarbons (α-Pinene, Camphene, β-Pinene, β-Myrcene, β-Cymene, β-Phellandrene, Limonene, Terpinolene, 3-Carene, α-Ocimene, γ-Terpinene, Aromandendrene), oxygenated monoterpenes (Linalool, Camphor, Borneol, Bornyl acetate, Eucalyptol), and sesquiterpene hydrocarbons (Aromadendrene, β-Caryophyllene, Humulene, Nerolidol).

GC-MS chromatograms were processed using PARADISe, a PARAFAC2 based deconvolution and identification system for direct analysis of complex raw GC-MS data (Johnsen et al., 2017). The targeted and untargeted data matrices are provided as Supplementary Material online (Supplementary Tables S1, S2). Triplicates were averaged and a number of samples did not have triplicates due to sample failure (sample 1, 2, 28, and 30). Compounds with retention times between α-Pinene (8.3 min) and Camphor (28.4 min) were retained for further analysis and compounds with zero or negative values were excluded.

### Statistical Analysis

All statistical analyses were performed using *R* statistical software (version 2.14.9). Initially untargeted data was converted into a Bray-Curtis similarity matrix and non-metric multidimensional scaling was performed using the Vegan package (Oksanen et al., 2013), which was visualized using ggplot2 (Wickham, 2016). A significant species effect on the untargeted chemical composition was tested using multivariate generalized linear modeling followed by analysis of variance (MGLM-ANOVA), which was performed using the manyglm and anova functions within the MVABUND package (Wang et al., 2012). Given the strong expected effects of species on plant chemistry (e.g., Hegnauer, 1962–1973; Rønsted et al., 2012), PERMANOVAs were ran to test for the effects of collection year, altitude and geography on untargeted chemical composition, using the adonis function with the Vegan package. Due to incomplete datasets, the effect of each was tested for individually in a PERMANOVA. Furthermore, sample GPS coordinates were converted into a principle coordinates of neighbor matrix (PCNM), with the resulting first two principle components (PCNM1 and PCNM2) used within the PERMANOVA testing for geographical effects on the untargeted chemical composition.

One-way analysis of variance (ANOVA) was performed to test for significant differences in untargeted chemical richness between species. Meanwhile, mixed linear modeling (MLM) was performed using the lmer function from the package lme4 (Bates et al., 2014). In these, the significance of random effects (altitude and geography (as PCNM1 and PCNM2) were determined using the drop1 function, which performed likelihood ratio tests (chi-square), whilst accounting for variation associated with the random effect (species).

The targeted dataset consisted of 20 compounds for which standards were obtained as described above (Table 2). The targeted chemical composition was analyzed as before, undergoing nMDS for visualization), MGLM-ANOVA and PERMANOVAs to assess for compositional effects, and ANOVA and MLM to assess for differences in chemical richness. Given that standards allowed for reliable quantification, each individual compound also underwent generalized linear modeling (GLM) to test for species effects, with compound serving as the independent variable and species serving as the dependent using the glm function of the native stats package of *R*. MLM was performed to test for the effects of year of collection, altitude and geography (as PCNM1 and 2) whilst accounting for potential species effects. Individual compounds from the targeted data served as the independent variable, species served as the random effect, and either year of collection, altitude, or PCNM1 and PCNM2 (together) serving as the fixed effect.

## Results

### Species Effects

**Figure 3:**
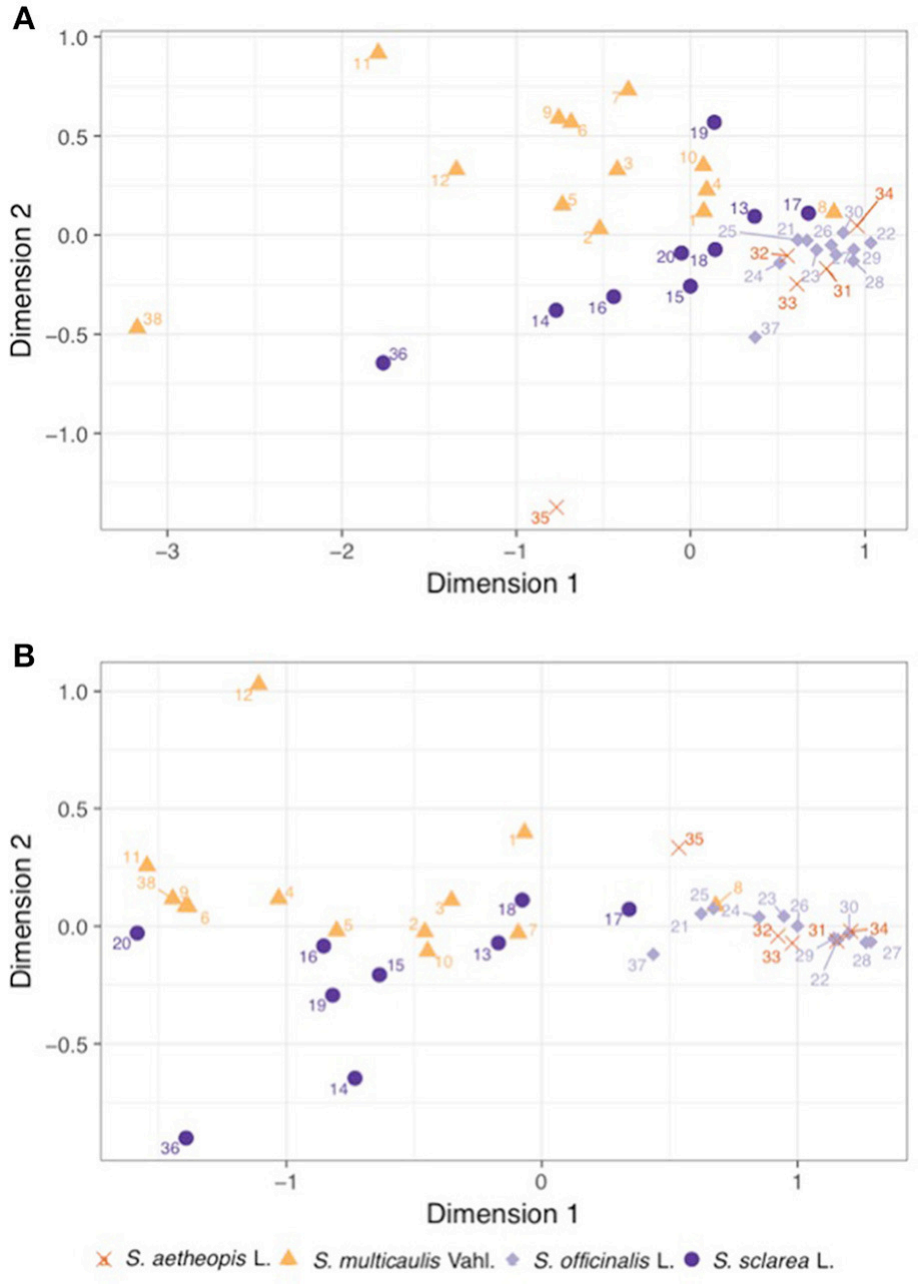
Bray-Curtis similarity matrices underwent non-metric multidimensional scaling using the **(A)** untargeted and **(B)** targeted chemical datasets in order to visualize them. The shape and color of the points on the plots represent different species, and demonstrate clear clustering in both datasets. Numbers of samples refer to the list of materials (Table 1).

After quality filtering a total of 285 compounds were within the untargeted dataset (Supplementary Table S2). Nearly all samples contained detectable levels of the majority of compounds, with untargeted chemical richness varying from 249 to 283 between samples, with no clear effect of species on the richness (ANOVA; df = 3, *F*-value = 1.16, *P*-value = 0.338). However, the untargeted chemical composition demonstrated clear clustering in the nMDS plot (Figure 3A), which proved highly significant (MGLM-ANOVA; Table 3).

**Table 3:** Tables of results for the untargeted and targeted chemical datasets.

**Untargeted dataset**					**Targeted dataset**				
**MGLM-ANOVA**					**MGLM-ANOVA**				
**Parameter**	**Df**	**Df difference**	***Deviance***	***P*-value**	**Parameter**	**Df**	**Df difference**	***Deviance***	***P*-value**
Species	34	3	**741.1**	**0.002**	Species	34	3	**649.8**	**0.002**
**PERMANOVA (Species as Strata)**					**PERMANOVA (species as strata)**				
**Parameter**	**Df**	***F*****-value**	***R***^**2**^	***P*****-value**	**Parameter**	**Df**	***F*****-value**	***R***^**2**^	***P*****-value**
**Year**	**1**	**3.275**	**0.083**	**0.021**	Year	1	2.700	0.070	0.272
Altitude	1	1.372	0.042	0.607	Altitude	1	1.788	0.055	0.417
PCNM1	1	1.884	0.056	0.139	PCNM1	1	1.956	0.058	0.244
PCNM2	1	0.741	0.022	0.910	PCNM2	1	0.935	0.028	0.964

*Initially, species effects were tested for using multivariate generalized modeling coupled with analysis of variance (MGLM-ANOVA). Secondly, PERMANOVAs were performed individually for year of collection, altitude and geography (as principal coordinates of neighbor matrix 1 and 2). Bold text represents significant effects (P < 0.050)*.

Within the targeted dataset, samples varied from 14 to 20 in the number of the 20 compounds analyzed, strongly differed between species (ANOVA; df = 3, *F*-value = 29.95, *P*-value = <0.001), with *Salvia multicaulis* and *S. officinalis* having significantly higher targeted chemical richness (19.7 and 19.4, respectively) than *S. sclarea* and *S. aethiopis* (15.6 and 16.0, respectively). Targeted chemical composition clearly clustered by species within the nMDS plot (Figure 3B), which was also highly significant (MGLM-ANOVA; Table 3).

**Figure 4:**
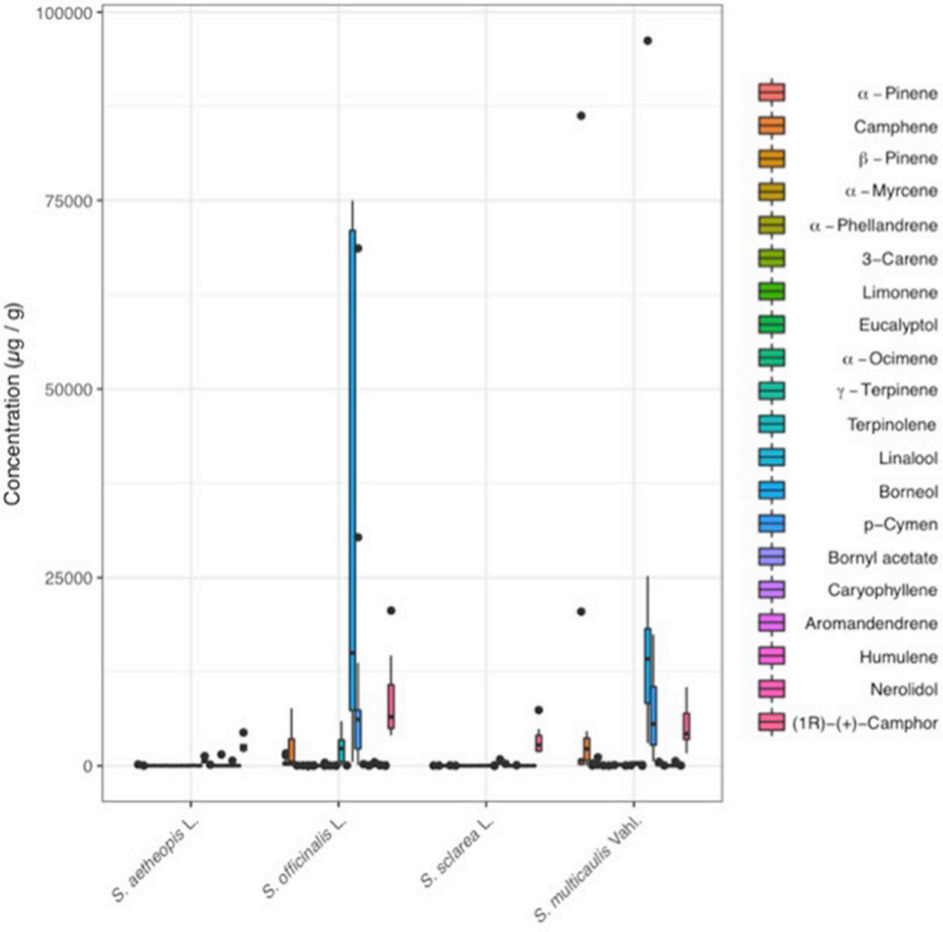
Boxplot for the 20 standard compounds analyzed within the targeted dataset. *Salvia officinalis* and *S. multicaulis* contained the highest concentrations of compounds, with borneol being the major compound in both species, and p-cymene, and camphor and α-pinene, in highest concentrations. *Salvia sclarea* had intermediate concentrations of the compounds, mainly in the form of camphor. *S. aethiopis* contained the fewest number of compounds, and when present, in the lowest concentrations.

Within the targeted dataset, *S. officinalis* and *S. multicaulis* consistently contained significantly higher concentrations when compared to the other two species (Figure 4). For example, borneol was the most abundant compound, ranging from 172 to 96,234 ng/μL, and were significantly higher in *S. officinalis* (31,954 μg/g) and *S. multicaulis* (21,964 μg/g) than averages in *S. sclarea* (436 μg/g), and *S. aethiopis* (675 μg/g) (Table 2). p-cymene was the second most abundant compound that was also concentrated in *S. officinalis* (11,559 μg/g) and *S. multicaulis* (6,774 μg/g) when compared to *S. sclarea* (51 ng/μL) and *S. aethiopis* (47 μg/g). Whilst camphor was present in lower abundance than borneol and p-cymene in *S. officinalis* (8291 μg/g) and *S. multicaulis* (5,273 μg/g), it was also present in comparable quantitates in *S. sclarea* (3,246 μg/g) and in two of the five *S. aethiopis* samples (average 2,696 μg/g). Terpinolene was also in abundance within *S. officinalis* (2,387 μg/g) whilst being almost absent from the other species. The other 15 compounds were present in relatively low amounts across all samples.

### Sampling Age Effects

**Figure 5:**
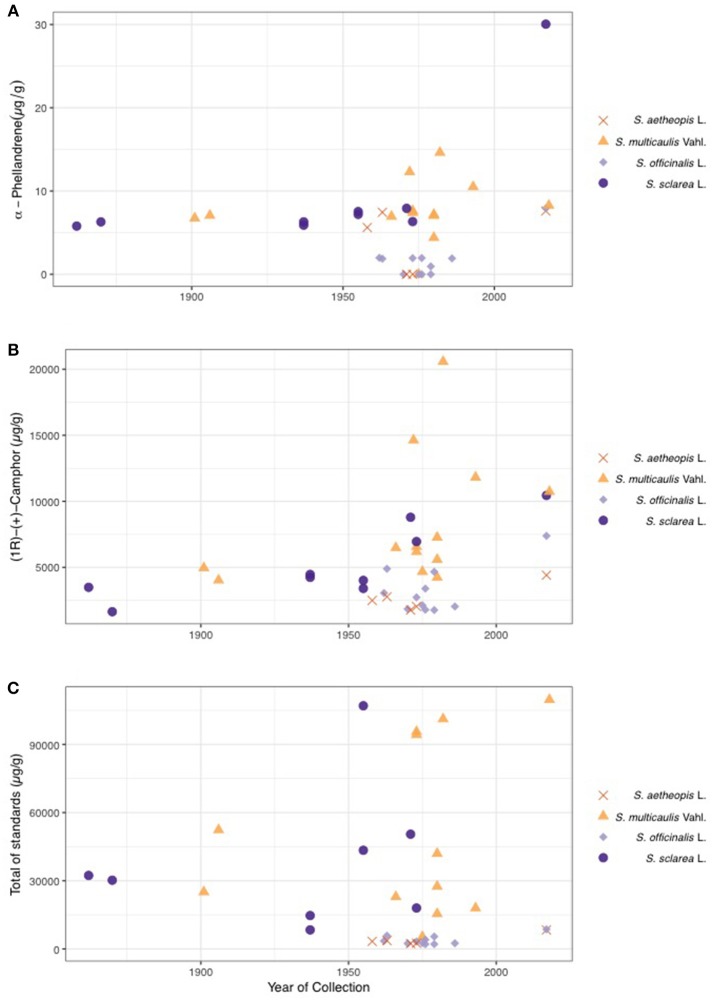
Plots illustrating concentration of α-phellandrene **(A)** and camphor **(B)** over time (year of collection). Both these and total chemical concentration of the 20 standards **(C)** significantly varied over the 150-year sampling period. The shape and color of the points on the graphs represent different species.

Given the confirmation of species effects on plant chemical composition, we next investigated age effects on the chemical composition. PERMANOVAs for both the targeted and untargeted datasets did not demonstrate a significant age effect on chemistry (PERMANOVA; Table 3). There were no significant effects of age of sample on either targeted or untargeted chemical richness (MLM; Untargeted χ_2_ = 3.79, *P*-value = 0.051 and targeted χ_2_ = 0.07, *P*-value = 0.787). Two individual compounds significantly declined in concentration over time (MLM; Table 4), α-phellandrene (Figure 5A), which was a very minor constituent, and camphor that was present in all four species (Figure 5B). Total amount of the compounds in the targeted dataset also significantly declined over time (MLM; χ_2_ = 4.11, *P*-value = 0.042) (Figure 5C).

**Table 4:** Generalized linear modeling was performed to test for significant species effects on each of the 20 standards constituting the targeted dataset.

**Compound**	***S. aethiopis***		***S. officinalis***		***S. sclarea***		***S. multicaulis***		**Sampling year**		**Altitude**		**PCNM1**		**PCNM2**	
	***T*-value**	***P*-value**	***T*-value**	***P*-value**	***T*-value**	***P*-value**	***T*-value**	***P*-value**	**Chi-Square**	***P*-value**	**Chi-Square**	***P*-value**	**Chi-Square**	***P*-value**	**Chi-Square**	***P*-value**
α-Pinene	0.006	0.996	0.122	0.904	0.000	1.000	2.638	0.013	4.954	0.026	3.697	0.055	2.888	0.089	1.461	0.227
Camphene	0.004	0.997	**4.141**	**<0.001**	0.003	0.998	**4.001**	**<0.001**	0.132	0.716	0.461	0.497	0.083	0.774	0.203	0.652
β-Pinene	0.422	0.676	0.804	0.427	0.329	0.744	**2.773**	**0.009**	4.539	0.033	3.626	0.057	2.450	0.118	1.146	0.284
α-Myrcene	0.189	0.851	0.378	0.708	0.272	0.787	2.500	0.017	4.344	0.037	3.607	0.058	2.483	0.115	1.171	0.279
α-Phellandrene	1.965	0.058	**5.909**	**<0.001**	1.189	0.243	**5.908**	**<0.001**	**6.852**	**0.009**	1.502	0.220	0.819	0.365	5.420	0.020
3-Carene	**9.491**	**<0.001**	**15.724**	**<0.001**	**11.539**	**<0.001**	**9.586**	**<0.001**	2.867	0.090	0.872	0.350	1.840	0.175	0.251	0.617
Limonene	0.000	1.000	**4.807**	**<0.001**	0.000	1.000	**3.171**	**0.003**	4.647	0.031	0.344	0.557	0.540	0.463	1.596	0.207
Eucalyptol	0.000	1.000	**9.727**	**<0.001**	0.000	1.000	**6.276**	**<0.001**	0.620	0.431	0.607	0.436	1.045	0.307	0.000	0.995
α-Ocimene	**7.728**	**<0.001**	**13.407**	**<0.001**	**9.748**	**<0.001**	**8.992**	**<0.001**	6.232	0.013	0.091	0.763	0.221	0.638	0.917	0.338
⋎-Terpinene	1.805	0.080	**3.528**	**0.001**	**2.126**	**0.041**	**3.898**	**<0.001**	4.204	0.040	1.569	0.210	0.581	0.446	1.255	0.263
Terpinolene	0.082	0.935	**7.423**	**<0.001**	0.068	0.946	0.767	0.448	0.535	0.464	0.069	0.792	2.395	0.122	0.787	0.375
Linalool	1.001	0.324	**3.427**	**0.002**	1.417	0.165	**3.123**	**0.004**	5.696	0.017	1.603	0.206	0.159	0.690	2.116	0.146
Borneol	0.066	0.948	**5.022**	**<0.001**	0.063	0.950	**2.872**	**0.007**	0.104	0.747	2.740	0.098	0.802	0.370	0.328	0.567
p-Cymen	0.009	0.993	**3.600**	**0.001**	0.015	0.989	1.756	0.088	0.328	0.567	0.200	0.655	0.212	0.645	0.729	0.393
Bornyl acetate	0.201	0.842	1.788	0.083	0.242	0.810	**6.492**	**0.000**	1.303	0.254	0.339	0.561	1.010	0.315	0.153	0.696
Caryophyllene	**3.012**	**0.005**	0.253	0.802	0.208	0.836	0.172	0.864	2.812	0.094	4.687	0.030	6.598	0.010	0.974	0.324
Aromandendrene	0.372	0.712	**2.968**	**0.005**	0.444	0.660	0.908	0.370	0.139	0.709	0.370	0.543	0.470	0.493	0.300	0.584
Humulene	2.185	0.036	0.584	0.563	0.115	0.909	1.524	0.137	6.485	0.011	5.711	0.017	3.687	0.055	2.088	0.148
Nerolidol	**4.401**	**<0.001**	**9.046**	**<0.001**	**5.422**	**<0.001**	**6.175**	**<0.001**	3.006	0.083	0.124	0.725	0.976	0.323	2.290	0.130
1R-(+)-Camphor	1.785	0.083	**8.851**	**<0.001**	**3.188**	**0.003**	**4.684**	**<0.001**	**9.184**	**0.002**	0.015	0.904	0.000	1.000	3.244	0.072

*Additionally, mixed linear modeling was performed individually for sampling year, altitude and geography (as principal coordinates of neighbor matrix 1 and 2). Bold text represents significant effects (P <0.050)*.

### Altitudinal and Geographical Effects

Altitude did not significantly affect the targeted or untargeted chemical composition (PERMANOVA; Table 3) and chemical richness (MLM; χ^2^ = 0.182, *P*-value = 0.670 and χ_2_ = 0.563, *P*-value = 0.453 in the untargeted and targeted datasets, respectively). Altitude also did not affect a single individual compound in the targeted dataset (MLM; Table 4). Geographic variation too did not affect chemical composition (PERMANOVA; Table 3) nor did it affect a single individual compound in the targeted dataset (MLM; Table 4). However, geographic variation significantly affected chemical richness in the untargeted dataset (PCNM1: χ_2_ = 8.37, *P*-value = 0.004, PCNM2: χ_2_ = 7.19, *P*-value = 0.007) but not the targeted (PCNM1: χ_2_ = 0.06, *P*-value = 0.794, PCNM2: χ_2_ = 3.34, *P*-value = 0.068).

## Discussion

### Terpenoid Composition of Salvia Species

The biological properties of essential oil of *Salvia officinalis* are attributed mainly to α-thujone and ß-thujone, camphor, and 1,8-cineole as reviewed by Raal et al. (2007). In the present study of herbarium samples, we also found monoterpenes to be the main fraction of compounds mainly consisting of borneol, p-cymene, camphor, and to a lesser extent camphene and terpinolene (Table 2). Whereas, high levels of borneol and camphor correspond to the findings of Raal et al. (2007), the high levels of p-cymene may reflect some degree of degradation of terpenoids, as p-cymene is often identified in aged oils (Turek and Stinzing, 2013). However, no age effect was observed for p-cymene.

The monoterpenes borneol, α-pinene, p-cymene, camphor, and camphene, were the main compounds found in *S. multicaulis* in the present study (Table 2). α-pinene, borneol, camphor, and camphene are also main compounds reported in other studies of *S. multicaulis* (e.g., Rustaiyan et al., 1999; Tepe et al., 2004; Morteza-Semnani et al., 2005; Bagci and Kocak, 2008; Karamian et al., 2013; Hatipoglu et al., 2016) in addition to 1,8-cineole, eucalyptol, bornyl acetate, and sesquiterpenes such as ß-caryophyllene and germacrene-D.

*Salvia sclarea* and *S. aethiopis* generally contained fewer compounds and in lower concentration than *S. officinalis* and *S. multicaulis*. In the survey of 45 Turkish *Salvia* species by Hatipoglu et al. (2016), both species were found to be rich in sesquiterpenes relative to monoterpenes. Our targeted dataset only included comparably few sesquiterpenes compared to monoterpenes. In *S. aethiopis*, we mainly found the oxygenated monoterpene camphor and lower amounts of borneol as well as the monoterpene hydrocarbon terpinolene and the sesquiterpenes ß-caryophyllene and α-humulene (Table 2). Other studies have primarily reported a range of sesquiterpenes from *Salvia aethiopis* including β-caryophyllene, bicyclogermacrene, germacrene-D, α-humulene, and α-copaene (e.g., Torres et al., 1997; Rustaiyan et al., 1999; Chalchat et al., 2001; Gulluce et al., 2006; Hatipoglu et al., 2016, and references therein). In *S. sclarea*, we found mainly the oxygenated monoterpenes camphor and borneol, which is in line with other studies reporting oxygenated monoterpenes such as linalyl acetate, linalool, and α-terpineol to be the main components (e.g., Gülçin et al., 2004; Farka et al., 2005; DŽamić et al., 2008; Ghani et al., 2010; Sharopov and Setzer, 2012). Previously, germacrene-D, β-caryophyllene, bicyclogermacrene sesquiterpenes have been reported from this species (Gülçin et al., 2004; Farka et al., 2005; DŽamić et al., 2008).

Among 108 volatile compounds across 45 Turkish *Salvia* species (Hatipoglu et al., 2016), both total yield and percentage of individual compounds were highly variable between species. α-pinene, camphene, β-pinene, 1,8-cineole, camphor, and borneol were the main chemical markers of monoterpene rich species, whereas β-caryophyllene, germacrene-D, β-bisabolene, bicyclogermacrene, caryophyllene oxide, and spathulenol were the main chemical markers in sesquiterpene rich species. In summary, several studies of essential oil components of different *Salvia* species have reported presence of a huge range of compounds, many in trace amounts, and other consistently present in higher amounts in specific species.

In the present study of herbarium specimens, we observed a significant species effect using both a targeted and an untargeted approach. Variation in study design including the number of compounds, geographical origin of samples, samples per species, and plant parts analyzed, makes it difficult to directly compare our findings with the literature. However, in general the number and amount of compounds as well as the main individual chemical compounds found in the present study are also reflected in the literature confirming that the species profiles we observe across 150-years of herbarium specimens are comparable to modern samples.

### Suitability of Herbarium Specimens for Extraction of Plant Metabolite Data

We observed a strong species effect and a weak geographical effect on terpenoid chemical composition of four *Salvia* species across the Mediterranean and no overall effect of sampling age, although two individual compounds did change with age of samples. These findings suggests that chemical composition appears to be well preserved in herbarium samples over at least 100–150 years suggesting herbarium samples can be used for chemical screening as well as for preliminary studies of pharmacological activity, which is also supported by previous studies reporting little effect of sampling age on overall chemical profiles (Eloff, 1999; Zangerl and Berenbaum, 2005; Cook et al., 2009; Yilmaz et al., 2012; Jafari et al., 2018). Herbarium samples may therefore provide an untapped resource for preliminary studies as well as for large-scale comparative surveys across taxonomical and geographical ranges. Whereas overall chemical composition appears to be well preserved over 100–150 years, the concentrations of individual compounds may be affected and we would therefore suggest herbarium samples may be well-suited for qualitative studies, whereas caution is needed in the use of herbarium samples for quantitative studies depending also on the type of compounds of interest (Rønsted et al., 2017). At a larger scale, a wide and thorough phytochemical screening of herbarium samples from all over the world could give us a better understanding also of chemotaxonomic issues, and comparison with climatic data would allow for testing of long-term environmental impact.

### Environmental Effect on Terpenoid Profiles

We observed a geographical effect on chemical richness in the untargeted but not in the targeted dataset of *Salvia* species across the Mediterranean. Geographic variation had no effect on chemical composition or on individual compounds. Russo et al. (2013) found chemical composition of *S. officinalis* essential oils to vary depending on environmental factors such as altitude, water availability and soil conditions in Italy. We did not observe an effect of altitude in the present study. However, our sampling covered a wide geographical range and therefore the potential effects of altitude alone may have been confounded by sampling across multiple ranges across such large scales as the Mediterranean. Future studies may disentangle geographical and environmental effects by more intensive sampling across single or multiple altitudinal gradients. However, it should be noted that sampling from herbaria is limited by the number of specimens available in the collection and herbarium specimens are mostly collected as individual specimens rather than representing populations, which limits the prospects of comparative environmental studies.

Many other studies have shown that the yield and chemical composition of essential oils varied with ecological factors and geographical areas (e.g., Uribe-Hernández et al., 1992; Salgueiro et al., 1997; Viljoen et al., 2006; Liu et al., 2015; Rezende et al., 2015; Jaradat et al., 2017). Essential oils play important biological functions related to environmental adaptation, protection against biotic and abiotic stresses, and pollinator attraction (Jassim and Naji, 2003; Zangerl and Berenbaum, 2005). Therefore, plants of the same species growing under different environmental conditions may differ in the composition of their essential oils in response to different environmental pressures (Weiss and Edwards, 1980; Salimpour et al., 2011). Over evolutionary time scales, unique chemotypes may eventually develop into separate genotypes adapted to different environmental conditions (Verpoorte et al., 2000; Heywood, 2002; Wink, 2003; Beccera, 2015). In addition to further comparison of environmental parameters, future studies may benefit from comparing genetic profiles of samples.

*Salvia* comprises nearly 1,000 species with distributions spread across the globe (Drew et al., 2017). Several *Salvia* species are used as food flavoring owing to their content of essential oils as well as in folk medicine for a range of conditions including microbial infections, inflammation, cancer, and malaria (Lu and Foo, 2002; Kamatou et al., 2008; Russo et al., 2013; European Medicines Agency, 2016; Hatipoglu et al., 2016).

Some species like *Salvia officinalis* are common and widespread, whereas others are narrow and potentially threatened endemics (Wood and Harley, 1989; Viney et al., 2006; Kahraman et al., 2012). A better understanding of the chemical composition of different *Salvia* species and how their chemical diversity is affected by environmental factors such as altitude can help inform both their local medicinal use as well as conservation policies and efforts of the threatened species.

## Conclusions

In the present study, freshly collected and herbarium samples of four species of *Salvia* were used to explore the effect of sampling age on terpenoid chemical profiles. Our results show that chemical profiles are primarily driven by a species effect and to a lesser extent geography. *Salvia multicaulis* and *S. officinalis* displayed higher abundance of most compounds than *S. aethiopis* and *S. sclarea*. Sampling age had no effect on overall chemical composition in the untargeted approach, and only a slight effect in the untargeted dataset. Two of twenty targeted compounds, α-phellandrene, and camphor, significantly declined with sampling year.

## Author Contributions

NR conceived the idea together with IF, CB, and RR. IF sampled the specimens. IF conducted the chromatography-mass spectrometry analysis together with TM and RR. IF analyzed the data together with CB. IF drafted the manuscript together with NR, CB, and RR. All authors read and approved the final manuscript.

### Conflict of Interest Statement

The authors declare that the research was conducted in the absence of any commercial or financial relationships that could be construed as a potential conflict of interest.

## Supplementary Material

Table S1 Filtered GC-MS data for all samples of the targeted dataset including occurrence of the 20 standard compounds.

Table S2 GC-MS results for all specimens in the untargeted dataset of 285 compounds identified tentatively by comparison with the NIST 8.0 library.
